# Association between preoperative hydration and cardiac surgery-associated acute kidney injury: a prospective observational study

**DOI:** 10.1186/s13019-026-04538-3

**Published:** 2026-07-23

**Authors:** Ghaith Mohsen, Julia Wallqvist, Zheng-Yii Lee, Anna Buckenmayer, Judith von Oppenkowski, Ajay Moza, Rachad Zayat, Daniel Catena, Lorenzo Germinarioa, Maren Kleine-Brueggeneya, Sascha Ott, Benjamin O’Brien, Markus Velten, Christian Stoppe

**Affiliations:** 1 Department of Cardiac Anesthesiology and Intensive Care Medicine, Deutsches Herzzentrum der Charité, Augustenburgerplatz 1, 13353 Berlin, Germany; 2 Charité-Universitätsmedizin Berlin, Corporate Member of Freie Universität Berlin and Humboldt- Universität zu Berlin, Charitéplatz 1, 10117 Berlin, Germany; 3 Department of Anaesthesiology, Intensive Care Medicine, Medical Faculty RWTH Aachen, University Hospital RWTH Aachen, Aachen, Germany; 4 Department of Anaesthesiology, Faculty of Medicine, Universiti Malaya, Kuala Lumpur, 50603 Malaysia; 5 Department of Anaesthesiology, Intensive Care, Emergency and Pain Medicine, University Hospital, Josef-Schneider-Strasse 2, 97080 Würzburg, Würzburg, Germany; 6 Department of Nephrology, Medical Clinic IV, Goethe-University, Frankfurt am Main, Germany; 7 Department of Cardiovascular Surgery, RWTH University Hospital Aachen, Aachen, Germany; 8 German Center for Cardiovascular Research (DZHK), partner site Berlin, Berlin, Germany; 9 Department of Anesthesiology, Outcomes Research Consortium, Cleveland Clinic, 9500 Euclid Avenue, Cleveland, OH 44195 USA; 10 Department of Perioperative Medicine, St Bartholomew’s Hospital and Barts Heart Centre, London, EC1A 7BE UK; 11 Department of Anesthesiology and Pain management, Division of Cardiovascular thoracic Anesthesiology, University of Texas Southwestern Medical Center, 5323 Harry Hines Blvd, Dallas, TX 75390 USA

## Abstract

**Background:**

Cardiac surgery associated acute kidney injury (CSA-AKI) is a common and serious complication following cardiac surgery with cardiopulmonary bypass (CPB), associated with prolonged hospitalization, increased costs, and higher mortality rates. Pathophysiological reasons and preventive strategies remain limited. Preoperative hydration and restriction of fluid fasting represent potentially modifiable factors, yet their association with postoperative CSA-AKI remains poorly understood.

**Objective:**

To explore the association between preoperative oral hydration and CSA-AKI in patients undergoing elective cardiac surgery with CPB.

**Design, setting, and participants:**

Prospective, single centre, observational hypothesis-generating study. Preoperative hydration surrogate was estimated based on patient self-reporting of fluid intake between admission and surgery. ROC analysis yielded a Youden Index-derived cut-off of 51.5 ml/h to stratify patients into low and high intake groups. The cut-off was derived within the study sample for exploratory stratification.

**Main outcomes and measures:**

Primary endpoint was incidence of CSA-AKI within seven postoperative days, defined according to KIDIGO creatinine criteria. Secondary endpoints included incidence of renal replacement therapy (RRT), postoperative renal function parameters, intensive care unit (ICU) and hospital length of stay (LOS), 30-day mortality, and postoperative complications. Secondary endpoints were considered exploratory.

**Results:**

Of the 92 patients analysed, 16,3% (15/92) of patients developed postoperative CSA-AKI. Using a data-derived expoloratory threshold of 51.5 ml/h, patients with lower preoperative fluid intake had a higher incidence of CSA-AKI thank patients with higher intake (33.3% [13/39]) vs. (3.8% [2/53]) (*p* < 0.001) respectively. RRT requirement was higher in the low-intake group (17.9% [7/39] vs. 0% [0/53]). The interval from last oral intake to induction of anaesthesia was significantly longer in the low-intake group (14.6 ± 5.1 vs. 11.5 ± 4.9, *p* = 0.004).

**Conclusions:**

Lower preoperative fluid intake was associated with higher observed rates of postoperative rates of CSA-AKI after applying a data-derived threshold. These exploratory data-driven findings and thresholds require prospective validation studies.

## Introduction

Cardiac surgery associated acute kidney injury (CSA-AKI) is a frequent complication, affecting more than 20% of adults undergoing cardiac surgery with cardiopulmonary bypass (CPB) [1, 2]. This complication increases the perioperative mortality eightfold, increases the length of critical care treatment and hospital length of stay [2, 3]. Even a mild postoperative increase in serum creatinine is associated with a marked rise in mortality [4–7]. Despite the seriousness of CSA-AKI, effective prophylaxis remains limited and pharmacologic strategies have produced variable results. Modifiable preoperative factors are therefore of particular interest.

Preoperative fluid balance plays an important factor in kidney protection, [8] and preoperative hypovolaemia is a biologically plausible trigger for renal injury, as it causes a reduction in the effective circulating volume and may reduce renal perfusion [9, 10]. This coincides with haemodilution, haemolysis, inflammatory reaction and vasopressor exposure during cardiac surgery and cardiopulmonary bypass (CPB) [1].

In spite of guidelines recommending patients may consume fluids up to or even less than two hours before aneasthesia, [11–14] prolonged fluid-fasting periods remain routine in many centres due to logistical delays, misunderstandings or old protocols [15, 16]. Extended fasting periods contribute to more discomfort, thirst and hypovolaemia, but its relevance for renal outcomes after cardiac surgery is not established.

Evidence from small randomised and observational studies suggests that preoperative fluid administration or hydration related markers may be associated with better postoperative renal outcomes in both cardiac and non-cardiac surgery [17, 18]. However, the relationship between a patient’s actual preoperative oral fluid intake, fasting duration, and postoperative renal outcomes after cardiac surgery is not well characterised and it is unclear whether sustained volitional oral hydration before surgery confers a renoprotective effect.

We hypothesise that lower self-reported preoperative oral fluid intake would be associated with an increased incidence of AKI and dialysis requirement in patients undergoing cardiac surgery. Therefore, we conducted a prospective observational study to investigate whether preoperative oral fluid intake as a surrogate for preoperative hydration influence the risk of AKI and dialysis after cardiac surgery with CPB.

## Methods

### Study design and setting

This was a single-centre, prospective, exploratory, hypothesis-generating observational pilot study at RWTH Aachen University Hospital, Germany. The protocol was approved by the institutional ethics committee (23 April 2019, No. 18 − 004 BOÄ). The trial is registered on Clinicaltrials.gov with the registration ID NCT03938181. No formal sample size calculation was performed. The target sample size was set at 100 patients based on feasibility. Therefore, the study should be interpreted as an exploratory pilot and was not powered for secondary outcomes. Written informed consent was obtained from all participants.

### Participants

Adult patients (> 18 years) undergoing elective cardiac surgery with CPB and cardioplegia between March and December 2019 were included.

Key exclusion criteria were pre-existing dialysis dependence, emergency surgery, preoperative i.v. fluid optimisation, preoperative mechanical circulatory support (LVAD, ECMO), pregnancy or lactation, and concurrent participation in other interventional studies. Patients who received contrast medium within 72 h preoperatively were also excluded.

### Data collection

Baseline variables included age, sex, body mass index, EuroSCORE, vital signs, comorbidities, left-ventricular ejection fraction, long-term medications, and preoperative laboratory values. Preoperative creatinine-based estimated glomerular filtration rate (eGFR) was calculated using the CKD-EPI (Chronic Kidney Disease Epidemiology Collaboration) Eq. [19].

Preoperative oral fluid intake, used as a surrogate for hydration was self-reported using patient diary completed daily from day of admission (or up to two days before surgery) until the day of surgery. Patients recorded each glass of water/soft drink/juice and each cup of tea/coffee. At the Institution, both the standard cup and glass have a volume of 150 ml, intake was therefore quantified as 150 mL per unit. Patients also documented the time of their last oral fluid intake before surgery.

Intraoperative data comprised operation characteristics, total fluids administered and urine output. Diary compliance was based on patient completion and was not externally validated by direct observation or objective hydration markers.

Postoperative data included fluid administration and urine output on postoperative days (POD) 0–2, vital signs, and laboratory values. Postoperative complications and ICU and hospital lengths of stay were recorded.

### Hypothesis

Exploratory aim and analysis plan: This prospective pilot study was designed to generate hypotheses regarding the association between preoperative hydration rates and CSA-AKI. As an exploratory step, we utilized ROC analysis and the Youden index to derive a data-driven cut-off value, stratifying patients into low and high-intake groups for descriptive comparison.

### Outcomes

Primary outcome: The study’s primary outcome was the incidence of CSA-AKI within the first 7 days after surgery, with AKI defined according to the KDIGO serum creatinine criteria [20]. Stage 1 AKI is defined as an increase in serum creatinine by at least 0.3 mg/dl during a 48-hour period or 1.5–1.9 x baseline within 7 days, Stage 2 as doubling the baseline creatinine level (on admission day), and stage 3 as tripling of the baseline serum creatinine level, or ≥ 4.0 mg/dl with an acute increase of at least 0.5 mg/d or the initiation of dialysis within the first 7 days.

Exploratory secondary outcomes: (1) Renal replacement therapy (RRT): initiation of any acute RRT modality (intermittent haemodialysis, sustained low-efficiency dialysis, or continuous therapies) during the first seven postoperative days. (2) renal trajectories: Evolution of serum creatinine and estimated glomerular filtration rate (eGFR) from baseline through postoperative day 2 and on day 7, calculated from routine laboratory measurements. (3) lengths of stay in the ICU and the hospital (4) 30-day all-cause mortality or in-hospital mortality, whichever occurred first; and (5) postoperative complications (infection, stroke, delirium, arrhythmia, and reoperation) occurring within 30 days or before hospital discharge.

### Perioperative clinical management

All patients were managed according to institutional standards to ensure consistent care. Protocolised elements included: antibiotic prophylaxis per cardiac-surgery guidelines; a standardized anaesthesia technique (balanced general anaesthesia with invasive monitoring; vasoactive use at clinician discretion within protocol); a uniform cardiopulmonary bypass (CPB) protocol (heparinization to target (activated clotting time) ACT, temperature management, cardioplegia strategy, and pump flows per institutional standards); and standardized postoperative ICU care, including ventilator weaning pathways, transfusion thresholds, and fluid management strategies (goal-directed fluids/vasopressors and diuretic use as clinically indicated). Criteria for initiating renal replacement therapy (RRT) followed the ICU’s nephrology protocol (refractory acidosis, electrolyte imbalance, fluid overload with organ dysfunction, or uremic complications).

### Statistical analysis

Analyses were performed in IBM SPSS Statistics v26 (IBM, Armonk, NY). Categorical variables are summarized as counts (percentages); continuous variables as mean ± SD (or median [IQR] when not normally distributed). Normality was assessed with Shapiro Wilk test. Between-group comparisons used independent samples t-tests for normally distributed data (with Levene’s test for homogeneity; Welch’s t-test if violated) and Mann-Whitney U tests if otherwise. Categorical variables were compared with Pearson’s χ² or Fisher’s exact test if > 20% of cells had expected count of < 5. Effect sizes are reported as φ for 2 × 2 tables and Cramér’s V for larger tables. Odds ratios (OR) and risk ratios (RR) with 95% confidence intervals were calculated where appropriate.

The average preoperative hydration rate (mL/h) was calculated as:

total recorded preoperative oral fluid volume (mL) from hospital admission to induction of anaesthesia ÷ elapsed hours in that interval (including fasting hours).

To explore thresholds, we constructed a receiver operating characteristic curve (ROC curve) for hydration rate and utilized the Youden index to derive a data-driven cut-off value, stratifying patients into low and high-intake groups for descriptive comparison. The resulting threshold was derived within the same dataset and was not externally validated.

Because of the small number of CSA-AKI events, no multivariable logistic regression was performed. All comparisons should therefore be interpreted as unadjusted, exploratory, descriptive and hypothesis generating.

Postoperative laboratory trajectories (e.g., serum creatinine, eGFR) were compared relative to baseline using repeated-measures ANOVA; Mauchly’s test assessed sphericity and Greenhouse-Geisser corrections were applied. Statistical significance was defined as *P* < 0.05 (two-sided).

## Results

### Patients

**Fig. 1:**
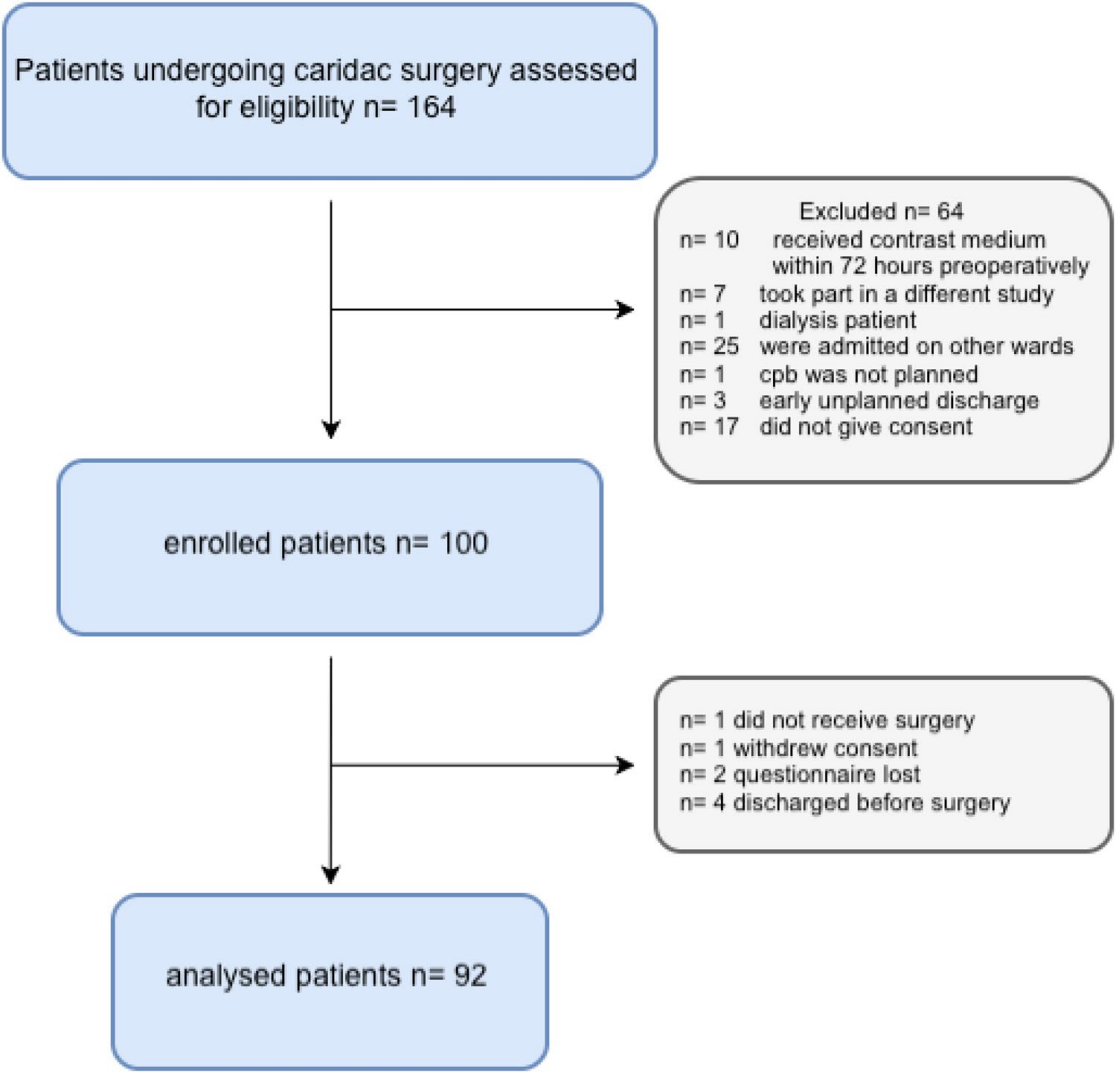
Flowchart of the study

A total of 164 patients were screened; 64 were ineligible, and 100 patients were enrolled, at which point recruitment ceased. Eight patients were subsequently excluded from the final analysis (one withdrew consent, one did not undergo surgery, four were discharged before surgery, and two had missing data), leaving a final cohort of 92 patients. (Fig. 1) The mean age of the study population was 67.4 ± 10.3 years, and 68.1% of patients underwent coronary artery bypass grafting. (Table 1)

**Fig. 2:**
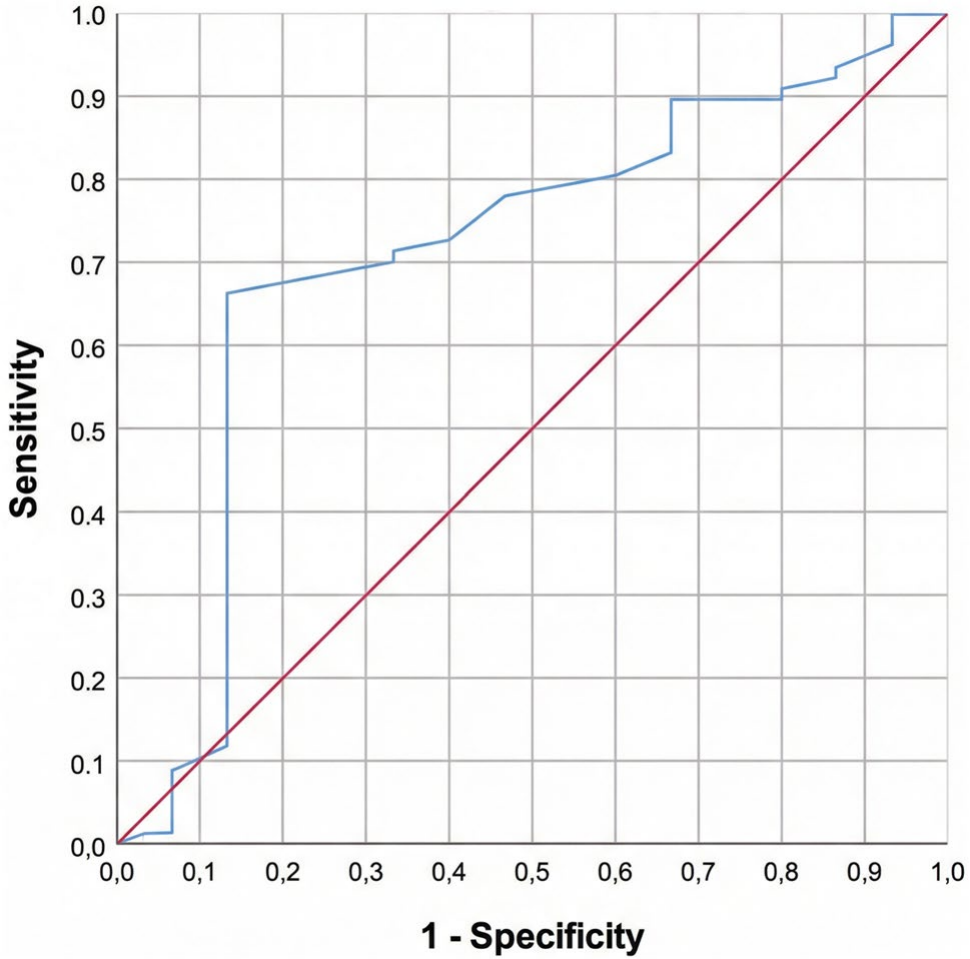
ROC Curve for hydration

For the entire cohort, the average preoperative oral fluid intake from admission to surgery was 58.2 ± 21.6 mL/h. To establish an exploratory threshold for patient stratification regarding postoperative AKI risk, a ROC analysis was performed (AUC 0.713). The Youden index (0.529) identified an optimal data-derived cut-off of 51.5 mL/h, which distinguished AKI probability with a sensitivity of 66.2% and a specificity of 86.7% (Fig. 2). Applying this threshold, patients were categorized into a high-intake group (*n* = 53; ≥ 51.5 mL/h) and a low-intake group (*n* = 39; < 51.5 mL/h). Consequent to this stratification, mean intake significantly differed between the cohorts (72.3 ± 16.2 mL/h vs. 38.9 ± 9.7 mL/h, respectively; *p* < 0.001).

The time from last oral intake to induction was significantly longer in the low-intake group with a mean interval of 14.6 ± 5.1 and a mean interval of 11.5 ± 4.9 in the high-intake group (*p* = 0.004).

### Preoperative fluid intake and development of AKI

Among the 92 patients included in the analysis, 15 (16,3%) developed postoperative AKI within the first seven postoperative days. The observed incidence was higher in the low-intake group. Because the cut-off was derived in this dataset, this comparison should be interpreted descriptively. AKI occured in 13 patients (33,3%) in the low-intake group compared with 2 patients (3,77%) in the high-intake group (*p* < 0,001). Table 2.

### Exploratory secondary outcomes

Renal replacement therapy (RRT).

Overall, 7/92 (7.6%) patients required postoperative RRT. All cases occurred in the low-intake group (7/39; 17.9%) and none in the high-intake group (0/53. *P* = 0.002). Renal function recovered in 6/7 patients by discharge. 1/7 progressed to dialysis-dependent chronic kidney disease at end of follow-up.Table 3.

Postoperative renal function.

**Fig. 3:**
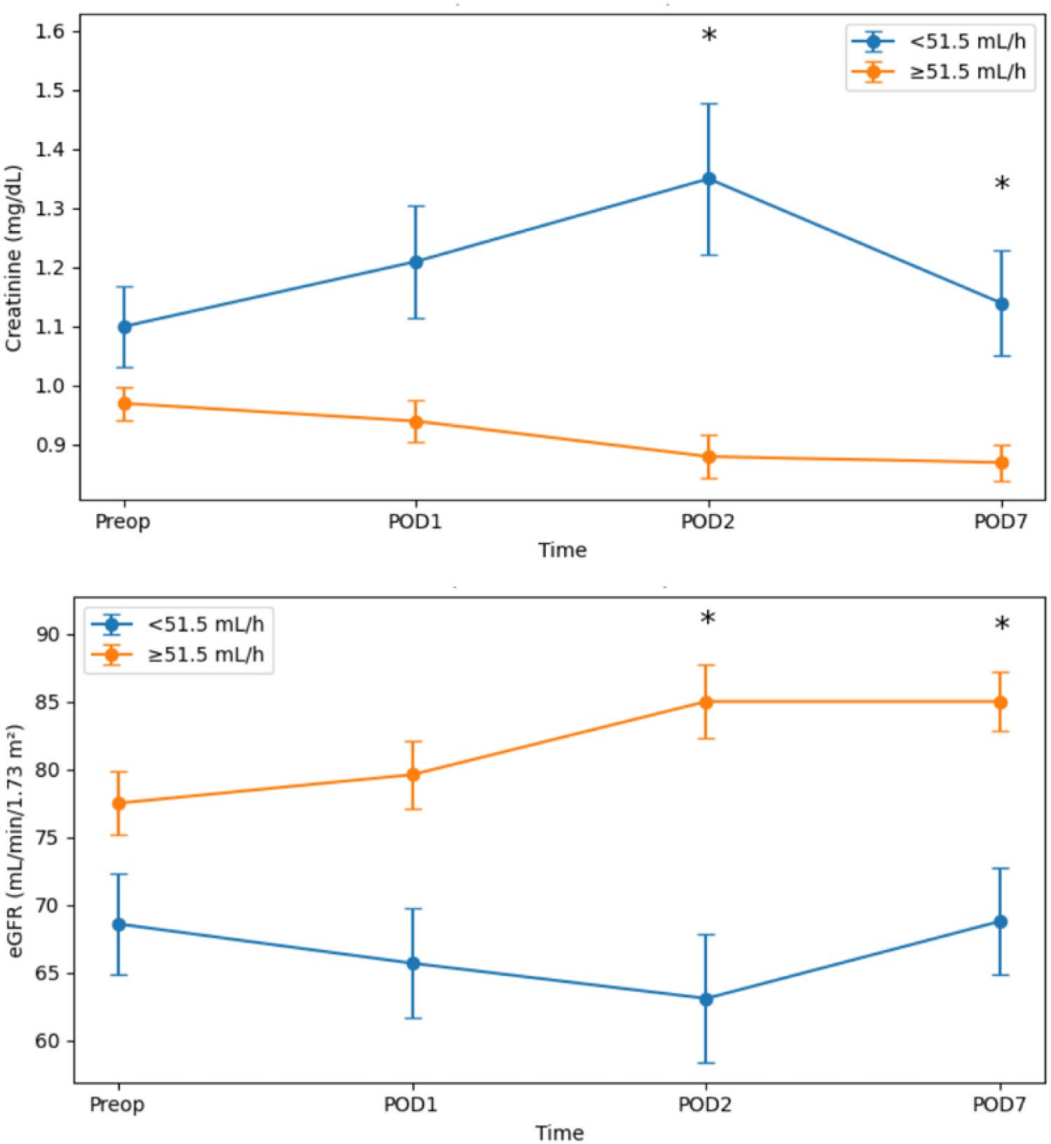
Creatinine and GFR courses until postoperative day 7

Preoperative creatinine and eGFR did not differ significantly between the groups. Postoperatively, peak serum creatinine was higher and eGFR lower in the low-intake group, with between-group differences becoming significant by postoperative day 2 (1.35 ± 0.8 vs. 0.88 ± 0.27 mg/dl *p* = 0.001; and 63.1 ± 29.4 vs. 85.0 ± 19.5 ml/min/1,73m^2^ *p* < 0.001 in the low vs. high-intake group, respectively) and persisting through day 7 (1.14 ± 0.55 vs. 0.87 ± 0.22 mg/dl *p* < 0.001; and 68.8 ± 24.8 vs. 85.0 ± 16.1 ml/min/1,73m^2^ *p* < 0.001) (Table 3; Fig. 3).

Length of stay.

ICU and hospital lengths of stay did not differ significantly between the groups with 16.8 ± 15.9 vs. 12.2 ± 8.8 days (*p* = 0.34) for hospital LOS and 156.8 ± 245.5 vs. 136.6 ± 229 h (*p* = 0.26) for ICU LOS in the low vs. high intake groups, respectively (Table 3).

Mortality and complications.

Thirty-day all-cause mortality was overall low with 1 patient dying in the low intake group (2.6%) and did not differ significantly between the groups. Rates of major postoperative complications. including infection, stroke, delirium, arrhythmia, reoperation, were not significantly different between groups (Table 3).

**Table 1:** Demographic, preoperative baseline characteristics of patients

	Low intake < 51.5 ml/h (*n* = 39)	High intake ≥ 51.5 ml/h (*n* = 53)	*P* value
Age	69.0 ± 11.8	66.3 ± 9.3	*0.08*
Male, n (%)	23 (59)	40 (75.5)	0.09
BMI (Kg/m2)	29.5 ± 5.6	27.7 ± 4.5	0.09
EuroScore mean ± SD	4.2 ± 4.2	3.4 ± 2.6	0.4
Heart rate mean ± SD	72.6 ± 12.9	70.5 ± 11.0	0.43
MAP mmHg mean ± SD	95.2 ± 9.8	94.8 ± 11.0	0.52
Previous cardiac surgery	0 (0)	1 (2.9)	0.39
COPD, n (%)	1 (2.6)	2 (3.8)	0.75
DM II, n (%)	14 (35.9)	12 (22.6)	0.17
Atrial fibrillation, n (%)	6 (15.4)	8 (15.1)	0.97
Neurologic impairment, n (%)	4 (10.3)	7 (13.2)	0.67
Arterial hypertension, n (%)	35 (89.7)	44 (83.0)	0.36
Nicotine abuse, n (%)	16 (41.0)	17 (32.1)	0.38
Alcohol abuse, n (%)	1 (2.6)	0 (0)	0.24
Peripheral arterial disease, n (%)	6 (15.4)	6 (11.3)	0.56
Ejection Fraction - ≥55, n (%) − 45–54, n (%) − 30–44, n (%) - <30, n (%)	29 (74.4)	43 (79.6)	0.44
	4 (10.3)	4 (7.4)	0.65
	6 (15.4)	6 (11.1)	0.57
	0 (0)	0 (0)	1.0
eGFR (mL/min/1.73 m²), mean ± SD	68.6 ± 24.0	77.5 ± 18.6	0.06
Creatinine (mg/dL), mean ± SD	1.1 ± 0.4	1.0 ± 0.2	0.39
Urea (mg/dL), mean ± SD	41.7 ± 16.1	35.9 ± 7.4	0.30
Creatine kinase (U/L), mean ± SD	108.3 ± 70.0	117.2 ± 82.9	0.58
Haemoglobin (g/dL), mean ± SD	13.3 ± 1.6	13.7 ± 1.8	0.15
Leukocytes (×10⁹/L), mean ± SD	7.9 ± 1.8	8.9 ± 10.2	0.21
CRP (mg/L), mean ± SD	8.7 ± 23.8	6.7 ± 11.3	0.4
Loop diuretics, n (%)	5 (12.8)	10 (18.9)	0.44
Thiazide diuretics, n (%)	8 (20.5)	8 (15.1)	0.5
Loop + thiazide diuretics, n (%)	1 (2.6)	2 (3.8)	0.75
Insulin, n (%)	6 (15.4)	6 (11.3)	0.57
Aspirin, n (%)	22 (56.4)	20 (37.7)	0.08
ACE inhibitors, n (%)	30 (76.9)	42 (79.3)	0.79
Calcium-channel blockers, n (%)	11 (28.2)	18 (34.0)	0.56
Beta-blockers, n (%)	32 (82.1)	34 (64.2)	0.06
Pre-Op fluid intake mL/h, mean ± SD	38.9 ± 9.7	72.3 ± 16.2	< 0.001
Time from Last oral intake to induction	14.6 ± 5.1	11.5 ± 4.9	0.004
Intra and postoperative characteristics			
Type of Surgery (%) - Bypass - Valve - Bypass and Valve	30 (76.9)	34 (64.2)	0.19
	8 (20.5)	17 (32.1)	0.22
	1 (2.6)	2 (3.8)	0.75
Duration of Surgery [min]	239.6 ± 78.2	228.0 ± 51.8	0.43
CPB Time [min]	105.5 ± 37.3	105.6 ± 30.9	0.1
Aortic Clamp Time [min]	66.1 ± 25.3	66.3 ± 18.2	0.8
Hypothermia < 32 °C (%)	34 (87.2)	50 (94.3)	0.23
Fluid Administration [ml]	2584.2 ± 1156.5	2187.7 ± 1114.1	0.09
Crystalloids [ml]	986.8 ± 513.2	1063.3 ± 778.8	0.92
Colloid [ml]	782.0 ± 1571.8	890.0 ± 1930.5	0.78
RBC Units [n]	2.3 ± 2.5	1.1 ± 1.2	0.03
Platelet Units [n]	1.1 ± 1.3	0.8 ± 1.2	0.23
Total Diuresis [ml]	1214.6 ± 906.6	1099.8 ± 562.8	0.97
Diuresis [ml/h]	281.2 ± 349.3	211.6 ± 141.7	0.67
Fluid intake 1. Postop day [ml]	4533.2 ± 1514.1	4849.3 ± 1810.8	0.46
Diuresis 1. Postop day [ml/h]	99.0 ± 46.5	107.0 ± 43.3	0.28
Fluid intake 2. Postop day [ml]	2598.0 ± 1480.2	2127.9 ± 1272.4	0.14
Diuresis 2. Postop day [ml/h]	95.1 ± 45.5	109.3 ± 39.3	0.19

Values are presented as mean ± SD or absolute numbers (%)

**Table 2:** Postoperative creatinine and GFR values

Parameter	Time point	< 51.5 ml/h (*n* = 39)	≥ 51.5 ml/h (*n* = 53)	*p*-value
Creatinine (mg/dl)	Pre Op	1.1 ± 0.4	1.0 ± 0.2	0.39
	Postop Day 1	1.21 ± 0.59	0.94 ± 0.26	0.07
	Postop Day 2	1.35 ± 0.80	0.88 ± 0.27	0.001
	Postop Day 7	1.14 ± 0.55	0.87 ± 0.22	< 0.001
eGFR (ml/min/1,73 m²)	Pre Op	68.8 ± 24.0	77.5 ± 18.6	0.06
	Postop Day 1	65,7 ± 25,1	79,6 ± 18,2	1,0
	Postop Day 2	63.1 ± 29.4	85.0 ± 19.5	< 0.001
	Postop Day 7	68.8 ± 24.8	85.0 ± 16.1	< 0.001

**Table 3:** Postoperative complications between the groups

Complication	< 51.5 ml/h (*n* = 39)	≥ 51.5 ml/h (*n* = 53)	*p*-value
Renal replacement therapy (%)	7 (17.9)	0 (0)	0.002*
Hospital LOS days mean (SD)	16.8 ± 15.9	12.2 ± 8.8	0.34
ICU LOS hours mean (SD)	156.8 ± 245.5	136.6 ± 229	0.26
Atrial fibrillation (%)	12 (30.8)	16 (30.2)	0.95
Delirium (%)	5 (12.8)	3 (5.7)	0.28
ARDS (%)	3 (7.7)	1 (1.9)	0.31
Stroke (%)	0 (0)	4 (7.6)	0.13
Reoperation (%)	2 (5.1)	6 (11.3)	0.46
Infection (%)	7 (18.0)	6 (11.3)	0.37
Death (%)	1 (2.6)	0 (0)	0.42*

*: p values were calculated using Fischer’s exact test

## Discussion

In this prospective observational study of patients undergoing cardiac surgery with cardiopulmonary bypass, lower self-reported preoperative oral fluid intake was associated with higher observed rates of postoperative kidney injury and RRT. Patients with lower fluid intake prior to surgery had a higher incidence of AKI and required dialysis more frequently compared with those with higher intake. these results suggest that preoperative hydration status is a potentially modifiable variable associated with kidney injury in this high-risk surgical population. Because the 51.1 ml/h threshold was derived from the same dataset and the analyses were unadjusted, these findings should be interpreted as an exploratory signal rather than evidence of causal effects. This warrants further investigation. Patients in the low-intake group received a higher number of RBC transfusions, this difference may be a statistical artifact of the small event numbers, however it may also reflect bleeding, CPB-related haemodilution and may represent a confounder or a mediator of postoperative AKI-risk. An additional finding was that the low-intake group had a significantly longer interval from last oral intake to induction. This suggests that lower preoperative oral hydration exposure may have reflected both reduced total fluid intake and prolonged preoperative fasting. This finding supports the possibility that avoidance of unnecessary prolonged fasting and implementation of structured clear-fluid protocols could improve preoperative oral hydration exposure. Our study extends the existing literature by providing prospective, real-world data from a cohort of cardiac surgery patients.

CSA-AKI remains a frequent and clinically important complication after cardiac surgery. As large trials of pharmacological and procedural approaches have yielded limited benefit [2]. There is continued interest in practical, modifiable perioperative factors that could reduce risk.

Only a few prior studies have specifically addressed preoperative hydration. Our study confirms the findings of Marathias et al., which demonstrated in a small randomized controlled trial that intravenous hydration with saline reduced the incidence of postoperative AKI and dialysis need in patients with chronic kidney disease undergoing cardiac sugery [17]. In contrast, Serrano et al. and Sarkar et al. reported no significant benefit of preoperative fluid substitution in patients undergoing major abdominal surgery or in diabetic patients undergoing cardiac sugery [21, 22]. However, no analysis examined the association between the amount of preoperative oral fluid intake and CSA-AKI.

The physiological plausibility of our findings is strong. Dehydration and hypovolemia reduce renal perfusion, diminish glomerular filtration and may exacerbate susceptibility to ischemia-reperfusion injury and inflammation associated with cardiac surgery [23–26]. Patients who begin surgery in a relatively hypovolemic state may have diminished physiological reserve to tolerate these additional insults.

In contrast, fluid overload leading to venous congestion can also impair kidney function by raising renal venous pressure, reducing the transrenal perfusion gradient and promoting interstitial edema [27]. Given the tight cardio–renal coupling in cardiac surgery patients, excessive preoperative volume loading may precipitate cardiac decompensation and secondarily worsen renal function [28]. Therefore, in cardiac surgery specifically, expert reviews have highlighted, although with limited evidence, adequate preoperative hydration and optimization of anaemia as promising renoprotective measures [8].

In spite of current ERAS [12] and ASA [13] guidelines recommendation, prolonged preoperative fasting remains common, driven by outdated practices, logistical delays and patient misconsception [29]. Continued oral fluid intake until the prescribed fasting interval is safe and may be beneficial. Our findings raise the hypothesis that adherence to fasting guidelines, coupled with structured preoperative hydration protocol, could reduce the incidence of CSA-AKI. Oral hydration is inexpensive, safe, and readily implemented. incorporation hydration status into perioperative risk assessment may improve patient stratification and guide preventive strategies.

This study is a single-centre pilot, and results may not generalise to other settings.

Moreover, the feasibility-based sample size and small number of events reduced statistical accuracy. Consequently, the study was not powered for secodary outcomes and type II error cannot be excluded. Preoperative intake data were self-reported. There was no formal compliance monitoring or validation against objective hydration markers, therefore reporing bias, recall bias and miscalculation cannot be excluded. Tea and coffee were included, but caffeine content and effects were not separately quantified. The total duration over which oral fluid intake was monitored was not reliably available for all patients. Although the interval from last oral intake to induction was available and differed significantly between the groups, fasting duration was not standardised and may have been influenced by operation schedule, institutional routines and patient’s clinical status and adherence. Therefore, prolonged fasting may represent a contributor to lower oral hydration. Furthermore, the fluid intake threshold (51.5 ml/h) was derived post hoc using ROC/Youden analysis within the same dataset and subsequently used for between-group comparisons. Therefore, p-values are likely optimistic, and the cut-off should be considered exploratory, sample-specific and not ready for clinical application. No internal or external validation was performed.

Additionally, analyses were primarily unadjusted; therefore, residual confounding by baseline risk, sex related differences in body composition and total body water, transfusion and perioperative management cannot be excluded, Because of the small number of AKI events, robust multivariable adjustment was not feasible without substantial risk of model overfitting. Notably, there were several non-significant baseline differences between the cohorts, with the higher hydration group being slightly younger and presenting with a higher baseline eGFR and lower prevelance of Diabetes. Because of these potential confounders, causal inference is not possible. The exposure definition (oral fluid intake rate from admission to induction) may also not reflect true physiologic hydration status. Objective measurement such ans IVC ultrasound, urine specific gravity or osmolality were not conducted. Futhermore, clinical and haemodynamic status may also have confounded the association. NYHA functional class, detailed right-ventricular function, quantative intraoperative vasopressor and inotrope exposure, duration of hypotension, CPB flow parameters, lactate and markers for low cardiac output syndrom were not systematically available. Finally, AKI was defined using creatinine criteria without urine output, and creatinine is a late and dilution-sensitive marker. These limitations underscore that the results are hypothesis-generating and require confirmation in independent cohorts and prospective interventional studies. A multicentre randomised controlled trial could compare standard care with a structured preoperative oral hydration protocol, potentially targeting the exploratory threshold identified here, while incorporating objective volume-status assessments, standardised fasting intervals, NYHA class, right-ventricular function, CPB perfusion variables and quantitative vasoactive and inotropic support. Sex and body composition should also be considered in future analyses, as absolute intake expressed as ml/h may not represent equivalent hydration exposure across patients with different body sizes and composition. Such trials should also assess optimal fluid volumes, timing, types of fluids and caffeine diuretic effects. Furthermore, integrating hydration and fasting parameters into perioperative risk assessment may enhance prediction of AKI and tailor interventions.

## Conclusion

In this single centre prospective pilot study, lower average preoperative oral fluid intake (as a surrogate for hydration rate) was associated with higher observed rates of CSA-AKI and RRT after cardiac surgery with CPB. Because the hydration threshold was derived post hoc within the same dataset, analyses were unadjusted, event numbers were small and oral intake may not equal physiologic hydration status, these findings should be interpreted as hypothesis-generation and require external validation before clinical application.

## Data Availability

No datasets were generated or analysed during the current study.
